# Transcranial magnetic stimulation set-up for small animals

**DOI:** 10.3389/fnins.2022.935268

**Published:** 2022-11-10

**Authors:** Jaakko O. Nieminen, Alexey S. Pospelov, Lari M. Koponen, Pauliina Yrjölä, Anastasia Shulga, Stanislav Khirug, Claudio Rivera

**Affiliations:** ^1^Department of Neuroscience and Biomedical Engineering, Aalto University School of Science, Espoo, Finland; ^2^BioMag Laboratory, HUS Medical Imaging Centre, University of Helsinki and Helsinki University Hospital, Helsinki, Finland; ^3^Biomedical Imaging Unit, A.I. Virtanen Institute, University of Eastern Finland, Kuopio, Finland; ^4^Faculty of Biological and Environmental Sciences, Molecular and Integrative Biosciences, University of Helsinki, Helsinki, Finland; ^5^Department of Clinical Neurophysiology, BABA Center, Children’s Hospital, Helsinki University Hospital and University of Helsinki, Helsinki, Finland; ^6^Neuroscience Center, Helsinki Institute of Life Science, University of Helsinki, Helsinki, Finland; ^7^Department of Physical and Rehabilitation Medicine, Helsinki University Hospital, Helsinki, Finland; ^8^INMED (INSERM U1249), Aix-Marseille Université, Marseille, France

**Keywords:** transcranial magnetic stimulation, TMS, coil, holder, electromyography, rat

## Abstract

Transcranial magnetic stimulation (TMS) is widely applied on humans for research and clinical purposes. TMS studies on small animals, e.g., rodents, can provide valuable knowledge of the underlying neurophysiological mechanisms. Administering TMS on small animals is, however, prone to technical difficulties, mainly due to their small head size. In this study, we aimed to develop an energy-efficient coil and a compatible experimental set-up for administering TMS on rodents. We applied a convex optimization process to develop a minimum-energy coil for TMS on rats. As the coil windings of the optimized coil extend to a wide region, we designed and manufactured a holder on which the rat lies upside down, with its head supported by the coil. We used the set-up to record TMS–electromyography, with electromyography recorded from limb muscles with intramuscular electrodes. The upside-down placement of the rat allowed the operator to easily navigate the TMS without the coil blocking their field of view. With this paradigm, we obtained consistent motor evoked potentials from all tested animals.

## Introduction

Transcranial magnetic stimulation (TMS) is a non-invasive brain stimulation method widely applied for clinical and research use in humans (Rossini et al., 2015). In small animals, e.g., rats and mice, TMS can be combined with invasive recording techniques. Rodent studies allow investigating, e.g., gene expression following repetitive TMS (Ru-Rong et al., 1998; Ljubisavljevic et al., 2015), mechanisms of neuroplasticity (Gersner et al., 2011), effects of brain stimulation in various disease models (Lu et al., 2015; Zhao et al., 2018; Legrand et al., 2019), and other effects of repetitive TMS (El Arfani et al., 2017; Cullen et al., 2019).

Administering TMS to a small rodent brain, however, has its problems (Wilson et al., 2018). First, inducing a sufficiently strong electric field in the cortex is difficult due to the small size of the brain compared to the size of the TMS coils (Weissman et al., 1992; Alekseichuk et al., 2019). Second, the small coils needed are subject to strong forces, which may cause problems in their mechanical integrity (Cohen and Cuffin, 1991; Yunokuchi and Cohen, 1991). Third, small coils are susceptible to heating (Wilson et al., 2018) and may require active cooling (Parthoens et al., 2016). Fourth, it is difficult to predict the spatial distribution of the TMS-induced electric field without a detailed computational model (Koponen et al., 2020). Despite the challenges, there is increasing interest in developing tailored TMS coils for rodents (Tang et al., 2016; Meng et al., 2018; Boonzaier et al., 2020; Cobos Sánchez et al., 2020; Pernia et al., 2020; Khokhar et al., 2021).

In this study, we developed an optimized figure-of-eight coil and a compatible holder to administer TMS on rats. The coil was designed to induce a given electric field strength with minimum energy, providing an optimal way to stimulate the rat brain. The holder, on which the rat lies upside down, was designed to allow navigating TMS without the coil blocking the field view of the operator. We demonstrate the TMS set-up with experiments on rats in which motor evoked potentials (MEPs) were recorded from limb muscles with needle electrodes.

## Materials and methods

### Transcranial magnetic stimulation coil

**FIGURE 1:**
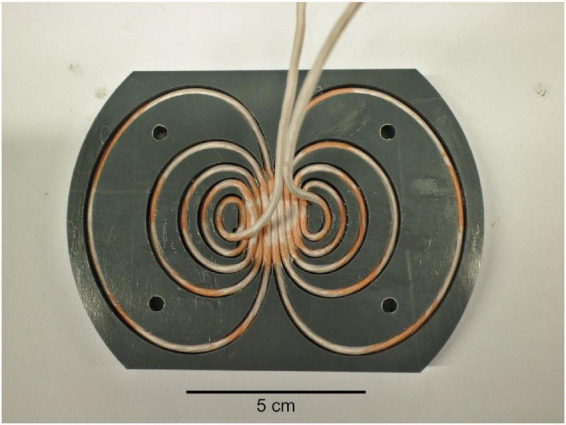
Photograph of the transcranial magnetic stimulation (TMS) coil. After the photograph was taken, the wires were glued with epoxy, and a cover was added.

We applied a convex optimization process (Koponen et al., 2017) to design a TMS coil for rodents. However, unlike in Koponen et al. (2017) where a coil was designed to work for the primary motor cortex of ten individual human head models, here the coil was designed to work on 35 cortical locations of one rat head model, which was assumed to result in a coil well-suited for stimulating on the superior parts of a rat brain. In the optimization, the rat head was modeled similarly to our earlier work (Koponen et al., 2020). As in Koponen et al. (2020), the 35 locations spanned a 15 mm by 10 mm region on the scalp, covering the superior parts of the cortex. No explicit focality constraints were applied; at each location, only the electric field in the brain at the point closest to the coil center was constrained. Thus, the electric field focality corresponded to the one that requires the least amount of energy to produce. The coil was further forced to be symmetric with respect to its two main axes. The animal imaging was approved by the University of Eastern Finland animal care committee and performed in accordance with their regulations and the guidelines of the European Community Council Directives 2010/63/EU. The coil dimensions were constrained to 116 mm by 84 mm, and the coil was manufactured from two polyvinyl chloride sheets similarly to Koponen et al. (2018a) with 1 mm between the bottom of the coil and the bottom of the lowest winding. The coil was wound with three layers of copper litz wire (70 circular 0.2-mm-thick strands, Rudolf Pack GmbH & Co. KG, Gummersbach, Germany) in series for a total of 15 turns of wire per wing (Figure 1). The coil inductance was approximately 9 μH and its resistance about 36 mΩ.

### Rat holder

**FIGURE 2:**
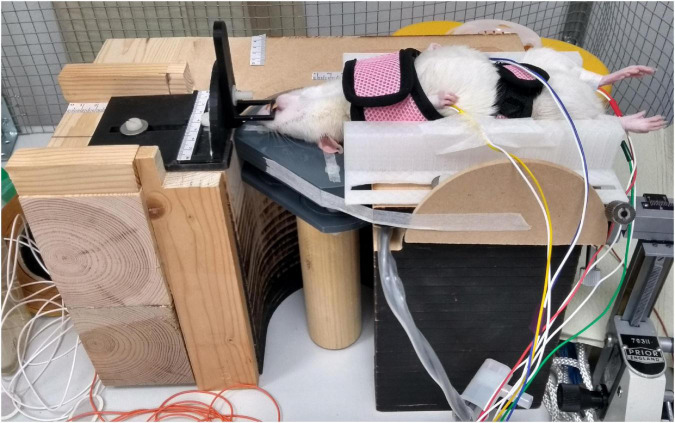
Experimental set-up. A rat is fixed upside down on the holder. The head of the rat on the surface of the transcranial magnetic stimulation (TMS) coil is kept fixed with the help of the tooth mount. Electromyography is recorded from the limbs with bipolar needle electrodes.

We constructed a holder to administer TMS on rats, with the rat head lying on the TMS coil (Figure 2). This configuration was preferred over the traditional one of holding a coil on the rat head, as the optimized coil is relatively large and would otherwise largely hinder the operator from observing the relative placement of the coil and the head. The body of the holder was made of wood and contains an 11.6-cm-diameter hole to fit our TMS coil and allow rotating the coil about its central axis. To keep the rat fixed, we 3D-printed a tooth mount and a small bed and integrated them into the body of the holder. The tooth mount consists of three pieces to control the head position in the rostral–caudal and lateral–medial directions while allowing rotating the head about the rostral–caudal axis. A rat walking harness (Trixie 61511 Harness for Small Animals for Rats Nylon, TRIXIE Pet Products, Inc., Fort Worth, TX, USA) was attached to the bed so that the body of the rat could be fastened securely and comfortably, while allowing the operator access to the limbs.

### Experiments

We followed the University of Helsinki guidelines for experimentation on rats. The details of the experimental procedures were approved by the national ethics committee for Animal Research (ESAVI/18276/2018). The experiments demonstrating the suitability of the developed coil for rodent studies were performed on young adult female Wistar rats (*N* = 4, weight 260–280 g). The animals were anesthetized (1.2 mg/g urethane) and fixed upside down on the holder (see Figure 2). The recording electrodes (bipolar, each made of two uninsulated 28G stainless steel needles) were implanted into the brachioradialis muscle of each forelimb and gastrocnemius muscle of each hindlimb (placement controlled by palpation of extended limbs) and fixed with tape. The ground electrode was implanted subcutaneously in the tail and fixed with tape. The quality of the recording was confirmed by an electrographic reaction to a paw pinch.

The TMS coil was driven by our custom stimulator electronics unit (Koponen et al., 2018a; Nieminen et al., 2022). The pulse waveforms were monophasic [60-μs rise time, 30-μs hold time, and 41.6-μs fall time for the current (Koponen et al., 2018b)]. The data were filtered (1.6–2,000-Hz bandpass filtering and a 50-Hz notch filter) and acquired with a 10-kHz sampling rate with a four-channel differential preamplifier connected to a four-channel amplifier (HS-4-D & UBA4-v7, BioAmp, Supertech Instruments, Pécs, Hungary), a data acquisition board (IX-408, iWorx Systems, Inc., Dover, NH, USA), and its software (LabScribe4, iWorx Systems, Inc.).

In the physiological trials, we varied the location and rotation of the TMS coil, as well as the applied current amplitude and direction. After the selection of the set of stimulation parameters, stimuli were administered in blocks of 10 with interstimulus intervals of 5–10 s to assess the reproducibility of the responses. The data were analyzed in MATLAB (R2018a, MathWorks, Natick, MA, USA). To deal with the TMS artifact, we first fitted a linear trend to the signals at 3–30 ms and subtracted it from the data at that interval. Then, we replaced the signal at 0–3 ms with a linearly interpolated waveform. Finally, the data were digitally filtered with a 100-Hz zero-phase, non-causal high-pass filter and visualized. As the aim of the current study was mainly to introduce the experimental set-up, the results were not quantified. The TMS intensity is reported relative to the maximum stimulator output (MSO) intensity, which in this study corresponded to 1-kV capacitor voltage.

## Results

In all four animals, further referred to as rats 1–4, the paw pinch caused a clear myographic response in the same limb. The maximal amplitude of the MEPs recorded later in each channel was comparable to the amplitude of these responses, verifying the quality of the recordings. Clear forelimb myographic responses to TMS were recorded in all four animals. Hindlimb responses were recorded in one animal (rat 4) at maximal MSO intensity.

**FIGURE 3:**
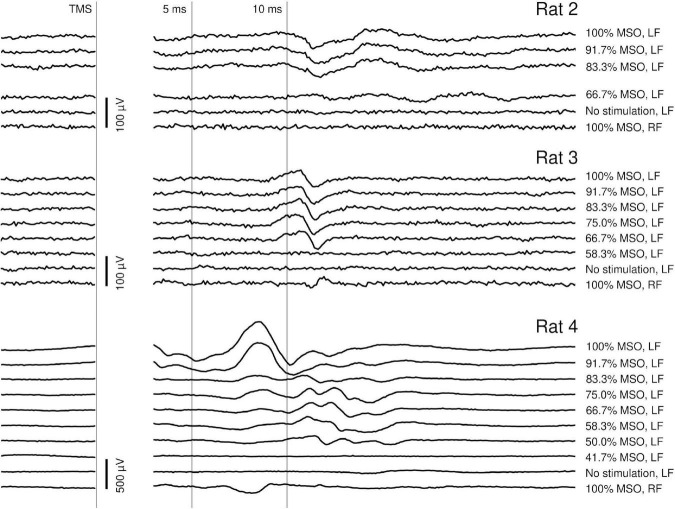
Dependency of the individual EMG responses on the transcranial magnetic stimulation (TMS) intensity at a fixed stimulation location in rats 2–4. MSO, maximum stimulator output; LF, left forelimb; RF, right forelimb. The bottom trace of every chart (100% MSO intensity, RF response) was recorded simultaneously to the top trace (100% MSO intensity, LF response), which allows assessing the lateralization of the response. In all three rats, stimulation below a specific intensity caused no responses. In rat 4, the response consisted of few components with different thresholds; a high-amplitude, high-threshold component had an onset latency of about 7 ms. This component was probably caused by a direct TMS action on subcortical or cervical motor structures. The component with a longer onset latency (9–10 ms) recorded in all rats was potentially caused by the activation of the cortex. The stimulation artifact has been masked.

**FIGURE 4:**
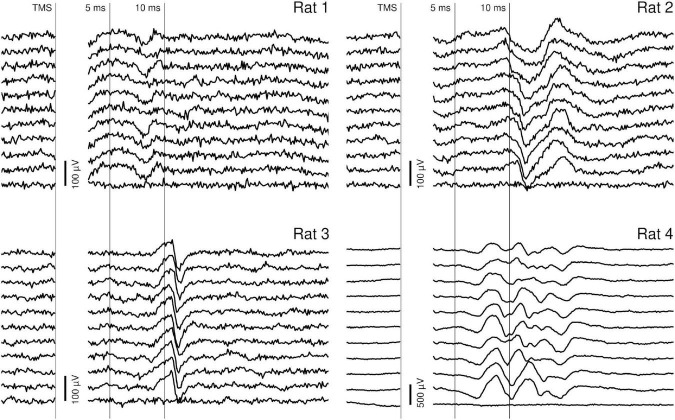
Stability of individual responses. Ten consecutive individual responses recorded within one block, with constants stimulation parameters. The traces were recorded from the right forelimb (rat 1) or from the left forelimb (rats 2–4). In each rat, the stimulation caused a highly reproducible EMG response with an onset latency of 7–10 ms and an offset latency of 10–15 ms. The stimulation artifact has been masked.

Typical myographic responses are presented in Figure 3. These responses had an onset latency of 7–10 ms and lasted up to 5 ms. In rats 1–3, the peak-to-peak amplitude of these responses was around 100 μV, while in rat 4 it reached 200–300 μV. Additionally, at high TMS intensities and often at more caudal stimulation sites, short-latency (2–7 ms), high-amplitude (up to 1 mV) responses were recorded (examples are presented in Figure 3; see the top traces recorded from rat 4). These responses were likely evoked by a direct effect of the TMS on cervical motor structures. In each rat, at a specific coil position, varying the TMS intensity resulted in highly stereotypical responses with clear thresholds. The responses were highly lateralized: while they were clearly seen in one forelimb, the other forelimb had no responses or had responses with much lower amplitude and different waveform. Stimulation with a constant position and intensity evoked responses with highly stable latency, amplitude, and waveform (see Figure 4).

## Discussion

In this study, we designed the TMS coil with a procedure that finds the optimal winding pattern on a given surface (Koponen et al., 2017). Compared to conventional tightly wound figure-of-eight or round coils, the windings of our optimized coil extend to a wider region (Figure 1) to minimize the energy needed to produce a given electric field profile in the brain without sacrificing the focality of the stimulation. Such optimized coils allow minimizing the heating of the coil, which ultimately limits the number of pulses that can be given before the coil overheats, and producing stronger stimuli with a given electronics unit. By limiting the coil size in the optimization, one may design more compact optimized coils, but this increases the needed stimulation energy. Similar optimization can be also applied to minimize the stimulus sound, which may be of interest if one wants to minimize auditory stimulation (Koponen et al., 2021). Our coil was designed to be planar. Optimized coils with windings lying on curved surfaces or surfaces extending around the head can be designed with similar procedures (Koponen et al., 2017; Cobos Sánchez et al., 2020). Coils extending around the head of a rodent (Cobos Sánchez et al., 2020) could be even more efficient than optimized planar or slightly curved coils (Koponen et al., 2015), but their manufacturing is more difficult. The methodology presented in this study and in the cited articles provides means for the experimenters to design TMS coils that best suit their applications and are compatible with their TMS power units. The custom stimulator electronics unit applied in this study (Koponen et al., 2018a; Nieminen et al., 2022) allows generating controllable pulse waveforms similar to, e.g., the device presented by Peterchev et al. (2014). As opposed to conventional TMS power circuits, the stimulator allows delivering monophasic TMS pulses with minimal energy losses. Despite the optimized design, applying repetitive TMS with long pulse trains would, however, benefit from an active cooling system for the coil.

The upside-down positioning of the rat on the coil allowed the operator to easily navigate the TMS administration without the coil blocking the field of view of the operator. The operator could easily see how the head is turned, tilted, and placed with respect to the coil. This configuration also simplified the mechanical support structures needed, as the coil, which is relatively heavy compared to the rat head, could stay fixed in the desired orientation on the table while the rat head was moved to achieve the intended TMS targeting. In addition, dorsal recumbency, giving access to the animal belly and extremities, opens new experimental settings not possible or difficult to perform with sternal recumbency. For example, this configuration could simplify the combination of biological sample collection (e.g., blood) and TMS in acute rodent disease models, e.g., models of ischemic stroke such as Middle Cerebral Artery Occlusion (MCAO). Indeed, an interesting future application of these procedures could be the monitoring of the MEP following reperfusion after transient MCAO. It can also simplify the experimental control of physiological parameters in the animal, e.g., breathing (intubation) and blood related parameters (artery or vein canulation). The access to abdomen can be useful for intraperitoneal injections. As the inverted position, however, is not natural for the animal, it may put additional pressure on the lungs, which may be of concern in experiments conducted under an anesthetics that involves a muscle relaxant such as medetomidine or xylazine. This arrangement is, however, commonly used in many acute physiological experimental procedures. When anesthetics are applied, and depending on the research question, one may need to take into account their effect on TMS-evoked brain activity. We further acknowledge that the upside-down positioning may not be compatible with all experimental needs but merely provides a further tool for the researchers when optimizing their set-ups.

In all four rats, TMS evoked clear EMG responses in the forelimbs, similar to what has been previously described in the literature (Rotenberg et al., 2010; Parthoens et al., 2016; Sykes et al., 2016; Tang et al., 2016; Boonzaier et al., 2020). Despite the similarity with previously performed detailed anatomical assessments (Kamida et al., 1998; Luft et al., 2001; Nielsen et al., 2007), it is difficult unequivocally to attribute specific components to the stimulation of particular brain structures. We, however, assume that the responses with the latency of 7–10 ms were likely triggered by the stimulation of the motor cortex, while the earlier responses may be related to subcortical structures, cervical motor structures, or a direct effect of the TMS pulse on muscles. The responses could not be due to movement artifacts because in many cases no visible movement accompanied the electrographic response.

The lateralization of the responses supports their cortical origin and indicates that the TMS coil can target specific motor areas. This observation is of value given that, in the coil design process, the focality of the electric field was not constraint explicitly, but only through the minimization of the energy required for the stimulation; when more focal electric fields are not needed, one should thus generally apply for the focality associated with the minimum-energy solution. Although at some coil positions and especially at high TMS intensities, multicomponent bilateral responses could be evoked, the traces simultaneously obtained from the forelimbs differed in the amplitudes and latencies of peaks, indicating activation of different brain areas.

Variability of the MEP waveforms between animals is likely to be related to the specifics of the recording configuration. Unlike concentric bipolar electrodes, the bipolar system consisting of two needles allows higher variability in the relative positions of active and reference electrodes. The stability of the MEP waveforms recorded from a single animal strongly supports this explanation: once the position of the recording electrodes was stable and the stimulation parameters were fixed, the MEPs were highly reproducible.

## Conclusion

The developed TMS set-up with an optimal TMS coil allowed eliciting specific motor evoked potentials in limb muscles of rats. The holder on which the rat was lying upside down allowed the operator to access the animal, without the TMS coil being on the way. Modern TMS coil optimization methods are expected to increase the efficiency of small-animal TMS.

## Data availability statement

The raw data supporting the conclusions of this article will be made available by the authors, without undue reservation.

## Ethics statement

The animal study was reviewed and approved by the National Ethics Committee for Animal Research (ESAVI/18276/2018).

## Author contributions

JN, AP, LK, PY, AS, and CR: conceptualization. JN, AP, and LK: software and visualization. JN and AP: data analysis and writing—original draft. JN, AP, PY, AS, and CR: investigation. All authors: methodology, resources, writing—review and editing, and approved the submitted version.
